# The human serum protein C4b-binding protein inhibits pancreatic IAPP-induced inflammasome activation

**DOI:** 10.1007/s00125-017-4286-3

**Published:** 2017-05-12

**Authors:** Klaudia Kulak, Gunilla T. Westermark, Nikolina Papac-Milicevic, Erik Renström, Anna M. Blom, Ben C. King

**Affiliations:** 1 Division of Medical Protein Chemistry, Department of Translational Medicine, Lund University, Inga Marie Nilssons Gata 53, Skåne University Hospital, S20502 Malmö, Sweden; 2 Department of Medical Cell Biology, Uppsala University, Uppsala, Sweden; 3 Department of Laboratory Medicine, Medical University of Vienna, Vienna, Austria; 4 Department of Clinical Sciences Malmö, Lund University Diabetes Centre, Lund University, Malmö, Sweden

**Keywords:** Amylin, Amyloid, C4BP, Complement, Diabetes, IAPP, Inflammasome, Inflammation

## Abstract

**Aims/hypothesis:**

Inflammasome activation and subsequent IL-1β production is a driver of islet pathology in type 2 diabetes. Oligomers, but not mature amyloid fibrils, of human islet amyloid polypeptide (IAPP), which is co-secreted with insulin, trigger NOD-like receptor pyrin domain containing-3 (NLRP3) inflammasome activation. C4b-binding protein (C4BP), present in serum, binds to IAPP and affects transition of IAPP monomers and oligomers to amyloid fibrils. We therefore hypothesised that C4BP inhibits IAPP-mediated inflammasome activation and IL-1β production.

**Methods:**

Macrophages were exposed to IAPP in the presence or absence of plasma-purified human C4BP, and inflammasome activation was assessed by IL-1β secretion as detected by ELISA and reporter cell lines. IAPP fibrillation was assessed by thioflavin T assay. Uptake of IAPP–C4BP complexes and their effects on phagolysosomal stability were assessed by flow cytometry and confocal microscopy. The effect of C4BP regulation of IAPP-mediated inflammasome activation on beta cell function was assessed using a clonal rat beta cell line. Immunohistochemistry was used to examine the association of IAPP amyloid deposits and macrophage infiltration in isolated human and mouse pancreatic islets, and expression of C4BP from isolated human pancreatic islets was assessed by quantitative PCR, immunohistochemistry and western blot.

**Results:**

C4BP significantly inhibited IAPP-mediated IL-1β secretion from primed macrophages at physiological concentrations in a dose-dependent manner. C4BP bound to and was internalised together with IAPP. C4BP did not affect IAPP uptake into phagolysosomal compartments, although it did inhibit its formation into amyloid fibrils. The loss of macrophage phagolysosomal integrity induced by IAPP incubation was inhibited by co-incubation with C4BP. Supernatant fractions from macrophages activated with IAPP inhibited both insulin secretion and viability of clonal beta cells in an IL-1β-dependent manner but the presence of C4BP during macrophage IAPP incubation rescued beta cell function and viability. In human and mouse islets, the presence of amyloid deposits correlated with higher numbers of infiltrating macrophages. Isolated human islets expressed and secreted C4BP, which increased with addition of IL-1β.

**Conclusions/interpretation:**

IAPP deposition is associated with inflammatory cell infiltrates in pancreatic islets. C4BP blocks IAPP-induced inflammasome activation by preventing the loss of macrophage phagolysosomal integrity required for NLRP3 activation. The consequence of this is the preservation of beta cell function and viability. C4BP is secreted directly from human pancreatic islets and this increases in response to inflammatory cytokines. We therefore propose that C4BP acts as an extracellular chaperone protein that limits the proinflammatory effects of IAPP.

**Electronic supplementary material:**

The online version of this article (doi:10.1007/s00125-017-4286-3) contains peer-reviewed but unedited supplementary material, which is available to authorised users.

## Introduction

Type 2 diabetes has an important inflammatory component [1] that drives insulin resistance in peripheral tissues and contributes to pancreatic islet dysfunction. The cytokine IL-1β plays an important role, and IL-1β-targeted therapy has shown promise in animal models [2] and clinical trials [3, 4]. IL-1β is produced by the inflammasome, where NOD-like receptor activation leads to formation of intracellular aggregates of apoptosis-associated speck-like protein containing a CARD domain (ASC) and pro-caspase 1, leading to caspase 1 activation and cleavage of pro-IL-1β to the mature active cytokine. Islet amyloid polypeptide (IAPP), which is co-secreted with insulin [5], forms oligomers that induce inflammasome activation [6]. In type 2 diabetes, IAPP and insulin secretion is increased to compensate for decreased insulin sensitivity in peripheral tissues, leading to increased local IAPP concentrations and formation of islet amyloid deposits. Over 90% of individuals with type 2 diabetes have detectable deposits at autopsy, and amyloid deposition correlates with beta cell loss [7]. Only low-molecular-weight IAPP species, not mature amyloid fibrils, cause inflammasome activation [6] or disrupt lipid bilayers [8, 9]. Destabilisation of the phagolysosomal membrane is the proposed mechanism by which IAPP and other particulates activate the inflammasome [6, 10].

Previously, we found that the serum protein C4b binding protein (C4BP) binds to human IAPP and co-localises to amyloid deposits in the pancreatic islets of individuals with type 2 diabetes [11]. Therefore, we decided to test the hypothesis that C4BP affects IAPP-induced inflammasome activation. Inflammasome activation by endogenous protein aggregates or amyloids is involved in many important diseases [12, 13] and identification of novel modes of inflammasome inhibition is therefore an important research goal.

## Methods

### Proteins

Plasma C4BP and recombinant monomeric C4BP α-chain were purified as described [14, 15]. C-terminal-amidated IAPP with C2–C7 disulfide bond was synthesised by J. I. Elliott at Keck Biotechnology (Yale University, New Haven, CT, USA) or by Cambridge Research Biochemicals (Billingham, UK), purified by HPLC and verified by mass spectrometry (>95% pure). IAPP with N-terminal Rhodamine B (RhB) conjugated by aminohexanoic acid linker was produced by Caslo ApS. IAPP was used as described [16]. C4BP was labelled with Alexa-Fluor 488 (A488) or pHrodo using amine conjugation kits (Invitrogen, Carlsbad, CA, USA).

### Cells

Cultured cells included THP1 cells (ATCC, Manassas, VA, USA), INS-1 rat insulinoma cells (a kind gift from C. Newgard, Duke University, Durham, NC, USA) [17] and primary human monocyte-derived macrophages (MDMs). See electronic supplementary material (ESM) Methods for further details. Islets and tissue from consenting human donors were provided by the Nordic Network for Islet Transplantation (Uppsala University, Sweden), under approval of the ethics committees at Uppsala and Lund Universities. Available data are reported in Tables 1 and 2.

**Table 1:** Available data for donors of pancreatic islets used for immunohistochemistry

Donor	Sex	Disease status	Disease duration (years)	Treatment
1	Male	T2D	16	Tolbutamide
2	Female	T2D	15	Chlorpropamide
3	Female	T2D	5	Chlorpropamide
4	Female	T2D	Unknown	Chlorpropamide
5	Female	T2D	26	Insulin

T2D, type 2 diabetes

**Table 2:** Available data for donors of isolated islets used for western blotting detection of C4BP

Donor	Sex	Age (years)	Disease status	Islet purity (%)
A	Male	71	T2D	86
B	Female	64	Non-diabetic	54
C	Male	65	Non-diabetic	86
D	Male	53	T2D	92
E	Male	68	Non-diabetic	91

T2D, type 2 diabetes

### Inflammasome activation assay

THP1 cells were plated with 250 nmol/l phorbol 12-myristate 13-acetate (Sigma, St Louis, MO, USA) for 2 h and allowed to differentiate overnight. Cells were primed with lipopolysaccharide (LPS) (Sigma) for 3 h, then OptiMEM medium (Thermo Fisher, Boston, MA, USA) containing inflammasome activators was added. See ESM Methods for further details.

### Western blot

Polyvinylidene difluoride membranes were blocked with fish gelatin (Norland products, Cranbury, NJ, USA) and then blotted with 1:5000 rabbit monoclonal anti-IL-1β (Abcam, Cambridge, UK, ab33774), 1:1000 polyclonal PK9008 rabbit anti-C4BP (in-house) or 1:50,000 mouse monoclonal anti-β-actin (Abcam). Supernatant fractions were first concentrated 20× using 3 kDa cut-off centrifugal filters (Millipore, Billerica, MA, USA). All blots were developed using 1:2000 horseradish peroxidase-conjugated secondary antibodies (DAKO, Glostrup, Denmark) and enhanced chemiluminescence reagents (Millipore). C4BP and IL-1β blotting specificity was determined by comparison with purified or recombinant proteins run in additional lanes.

### Cytokine detection

Secreted cytokines were detected using ELISA or HEK-Blue reporter cells and caspase-1 was detected using a Caspase-Glo assay. See ESM Methods for further details.

### Thioflavin T assays

IAPP monomers dissolved in hexafluoroisopropanol [16], or dissolved in DMSO and incubated 37°C for 2 weeks, were diluted into OptiMEM medium in the presence of 20 μmol/l thioflavin T (ThT) and either C4BP or BSA. Fluorescence (excitation 442 nm, emission 482 nm) was read every 10 min for 10 h in a CytationV multi-reader (BioTek, Winooski, VT, USA) at 37°C.

### Electron microscopy

During ThT assays, samples (1–10 μl) were removed and placed on formvar-coated Cu grids. Excess liquid was removed and the material was negatively contrasted with 2% (wt/vol.) uranyl acetate in 50% (vol./vol.) ethanol. For immunogold staining, samples were placed on formvar-coated Ni grids. IAPP was detected with rabbit antiserum raised against IAPP 1–37 and visualised with 10 nm gold particles. C4BP was detected with in-house mouse anti-human C4BP MK104 [18], visualised with 5 nm gold particles. Images were obtained at 75 kV using a 7100 electron microscope (Hitachi Medical Instruments, Tokyo, Japan).

### IAPP–C4BP uptake assays

MDMs or THP1 cells were incubated with labelled IAPP or C4BP, then harvested and uptake assessed by flow cytometry. Alternatively, internalisation was assessed by confocal microscopy, co-localising with Lysotracker dyes (Thermo Fisher). See ESM Methods for further details.

### Acridine Orange staining

MDMs were incubated in M-SFM with hydroxychloroquine (50 μg/ml; Sigma), IAPP (20 μmol/l) and C4BP (0.6 μmol/l) for 8 h, then stained with 1.5 μg/ml Acridine Orange (Sigma) for 15 min, washed twice and harvested for flow cytometric analysis.

### Viability and insulin secretion

INS-1 cells were incubated with conditioned supernatant fractions from activated or unactivated THP1 cells or with medium alone and cell death was measured by flow cytometry. Insulin secretion in high and low glucose conditions was measured by ELISA (Mercodia, Uppsala, Sweden). See ESM Methods for further details.

### Quantitative PCR

RNA was purified from cells and tissue samples by phenol–chloroform extraction. RNA quality was validated using a Biodrop spectrophotometer and Experion RNA electrophoresis analysis (Bio-Rad, Hercules, CA, USA). Reverse transcription was carried out using oligo-dT primers and quantitative PCR was performed using specific TaqMan assays (Thermo Fisher) for *CD59*, *C4BPA* and *IL1B*, using hypoxanthine-guanine phosphoribosyltransferase and β-2 microglobulin as reference genes, using a Viia7 Real-Time PCR system (Thermo Fisher). Results were calculated using the ∆∆C_t_ method.

### Islet staining

Pancreas sections from human donors and human IAPP (hIAPP) transgenic mice were stained for macrophages using CD68/SR-D1 antibody (Novus Biologicals, Littleton, CO, USA), or using guinea pig anti-insulin (DAKO) mouse anti-C4BP, sheep anti-pancreatic polypeptide (Bio-Rad) or Congo Red. See ESM Methods for further details.

### Statistical analysis

Statistics were calculated using GraphPad Prism 6 (GraphPad Software, La Jolla, CA, USA). In all figures, *p < 0.05, **p < 0.01 and ***p < 0.001, by ANOVA or two-way ANOVA where appropriate, with Bonferroni post hoc test. All data show means ± SD of three independent repeats, unless otherwise noted.

## Results

### C4BP inhibits IAPP-mediated IL-1β production

**Fig. 1:**
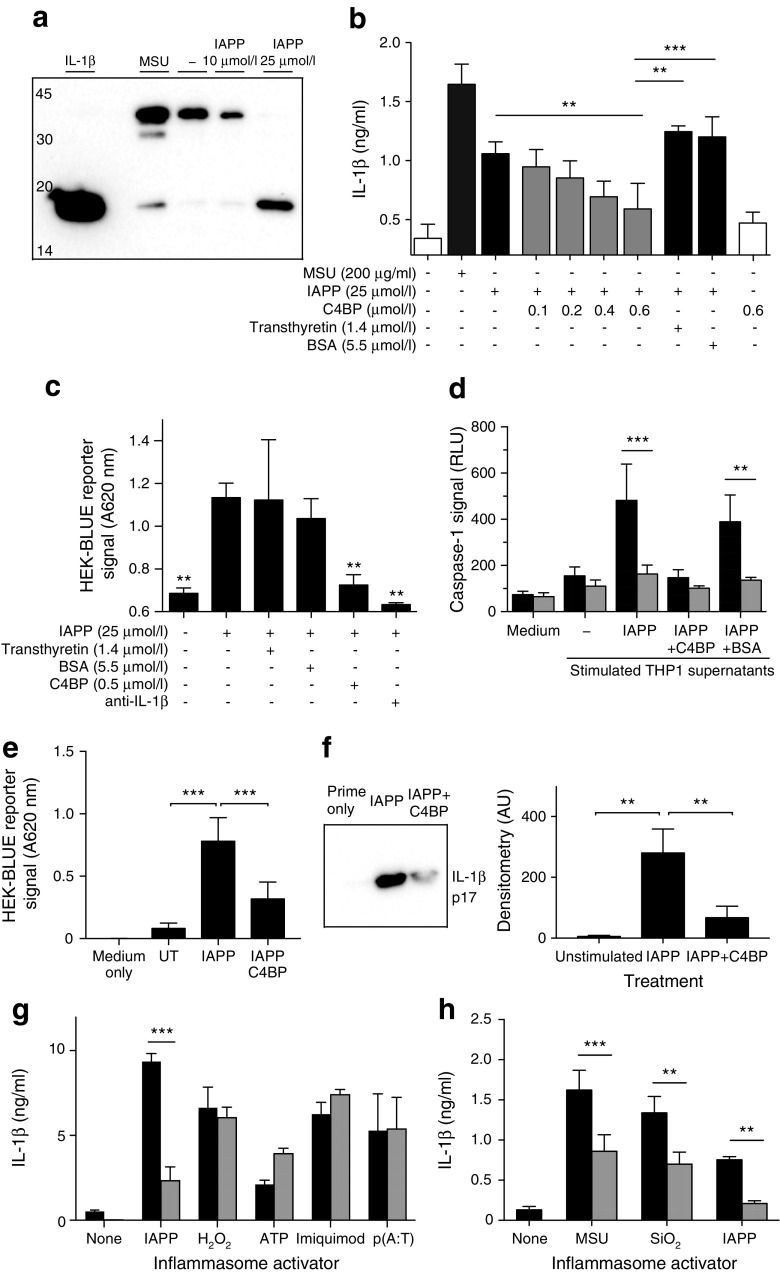
C4BP inhibits IAPP-mediated inflammasome activation. (**a**) Western blot for IL-1β in THP1 supernatant fractions, using MSU as positive control for inflammasome activation. Mature IL-1β, 17 kDa. (**b**) IL-1β ELISA of THP1 supernatant fractions stimulated with IAPP, in the presence of C4BP or control proteins. (**c**) HEK-BLUE reporter cell detection of IL-1β in THP1 supernatant fractions (A, absorbance). Anti-IL-1β, neutralising antibody. (**d**) Caspase-1 activity in stimulated THP1 supernatant fractions (black bars), with specificity verified using specific caspase-1 inhibitor Ac-YVAD-CHO (grey bars). RLU, relative light units. (**e**, **f**) C4BP also inhibited IAPP-mediated IL-1β release from MDMs (**e**) and primary monocytes (**f**), measured by HEK-BLUE detection and western blot/densitometry, respectively. (**g**) C4BP (0.5 μmol/l) inhibits IL-1β release by IAPP but not H_2_O_2,_ ATP, imiquimod or poly(dA:dT) [p(A:T)], as detected by HEK-BLUE reporter cells. Black bars, BSA-treated; grey bars, C4BP-treated. (**h**) C4BP addition (grey bars) also inhibited IL-1β secretion from THP1 cells stimulated with MSU or silica nanoparticles. Statistics in (**c**), compared with IAPP alone. AU, arbitrary units. **p < 0.01 and ***p < 0.001 as indicated; or compared with IAPP alone in (**c**)

Addition of IAPP to primed THP1 cells resulted in the processing of 35 kDa pro-IL-1β and secretion of 17 kDa mature IL-1β, detected by western blot (Fig. 1a). Addition of human plasma-purified C4BP, but not control proteins, reduced secretion of IL-1β to background levels, as determined by ELISA (Fig. 1b). This decrease in IL-1β secretion was confirmed using a reporter cell line specific for bioactive IL-1β (Fig. 1c). As confirmation of inflammasome inhibition, C4BP co-incubation reduced IAPP-induced caspase-1 activity in THP1 cells down to background levels (Fig. 1d). The effects on IL-1β secretion were also confirmed in primary MDMs and peripheral blood monocytes (Fig. 1e, f). C4BP was unable to inhibit inflammasome activation by ATP, H_2_O_2_ or imiquimod (which also activate the inflammasome via NOD-like receptor pyrin domain containing-3 [NLRP3]) or by poly(dA:dT) (which activates the inflammasome via absent in melanoma 2 [AIM2]) (Fig. 1g). However, C4BP was able to inhibit inflammasome activation by monosodium urate (MSU) crystals and silica nanoparticles (Fig. 1h), which, like IAPP, also cause NLRP3-dependent inflammasome activation via phagolysosomal membrane destabilisation [19].

### C4BP inhibits IAPP fibrillation

**Fig. 2:**
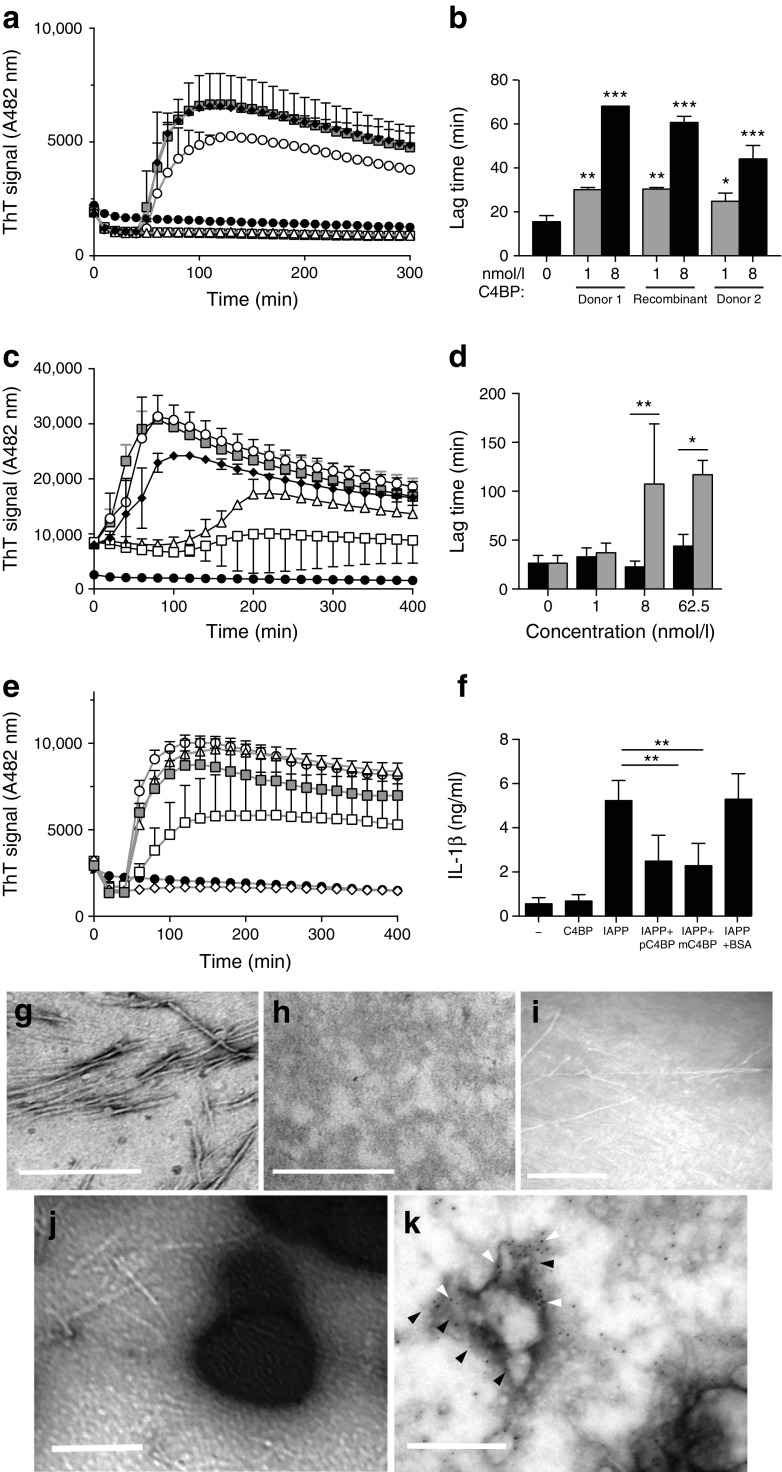
C4BP inhibits IAPP amyloid formation in THP1 cells under cell culture conditions. (**a**) ThT assay of 10 μmol/l hexafluoroisopropanol-dissolved IAPP monomers under cell culture conditions with C4BP or BSA (A, absorbance). (**b**) Lag-time extension after addition of plasma-purified C4BP or recombinant C4BP. (**c**) C4BP inhibits fibrillation of 4.25 μmol/l IAPP dissolved in DMSO added to cell culture medium. (**d**) Lag times from (**c**). Black bars, BSA-treated; grey bars, C4BP-treated. (**e**) Recombinant mC4BP inhibits IAPP fibrillation. (**f**) mC4BP also inhibits IAPP-mediated THP1 cell IL-1β production, similarly to polymeric recombinant C4BP (pC4BP). (**g**) Electron micrograph of IAPP fibrils formed after 2 h in the absence of C4BP. (**h**) No fibrils are formed after 2 h in the presence of C4BP. (**i**) Amyloid fibrils formed after 24 h incubation without C4BP. (**j**) Fibril formation after 24 h in the presence of 64 nmol/l C4BP. (**k**) Immunogold detection of IAPP (10 nm particles, white arrowheads) and C4BP (5 nm particles, black arrowheads) after 10 h of incubation. Scale bars, 200 nm. In (**a**), (**c**) and (**e**): grey squares, IAPP only; black circles, no IAPP; white circles, white triangles, white squares, white diamonds, C4BP added at 1, 8, 62.5, 500 nmol/l, respectively; black diamonds, BSA 62 nmol/l. *p < 0.05, **p < 0.01 as indicated and ***p < 0.001 as indicated; or compared with no added C4BP in (**b**)

ThT assays were used to assess the effect of C4BP on IAPP fibrillation in THP1 cells. Under cell culture conditions (OptiMEM medium, 37°C), C4BP significantly inhibited amyloid fibril formation (Fig. 2a) at nmol/l concentrations. This was consistent for recombinant C4BP and plasma-purified C4BP from various donors (Fig. 2b). Previously, using peptide from a different supplier under different reaction conditions, we found that C4BP enhanced fibrillation of IAPP diluted from DMSO-dissolved stocks [11] but we now found that under cell culture conditions C4BP strongly inhibited fibril formation from IAPP prepared in this manner (Fig. 2c, d). Recombinant C4BP α-chain monomers (monomeric C4BP [mC4BP]) also inhibited IAPP fibrillation, although higher molar concentrations were required (Fig. 2e). When added at equal μg/ml concentrations, mC4BP was equally effective as recombinant polymeric C4BP at inhibiting IAPP-mediated IL-1β release (Fig. 2f). To check whether C4BP inhibited fibrillation rather than blocking ThT binding, samples were removed from ThT assays and assessed by electron microscopy. Fibrillar aggregates were visible from 2 h onwards (Fig. 2g), whereas on addition of 64 nmol/l C4BP no fibrils were identifiable (Fig. 2h) for up to 10 h. After 24 h incubation, amyloid fibrils were present with or without C4BP (Fig. 2i, j). Fibrils formed in the presence of C4BP were associated with dark aggregates, which appeared to be C4BP deposits in the dried samples. Immunogold detection showed that C4BP and IAPP co-locate to amorphous aggregates at a 10 h time point, in the absence of fibrils (Fig. 2k).

### C4BP does not inhibit inflammasome priming

**Fig. 3:**
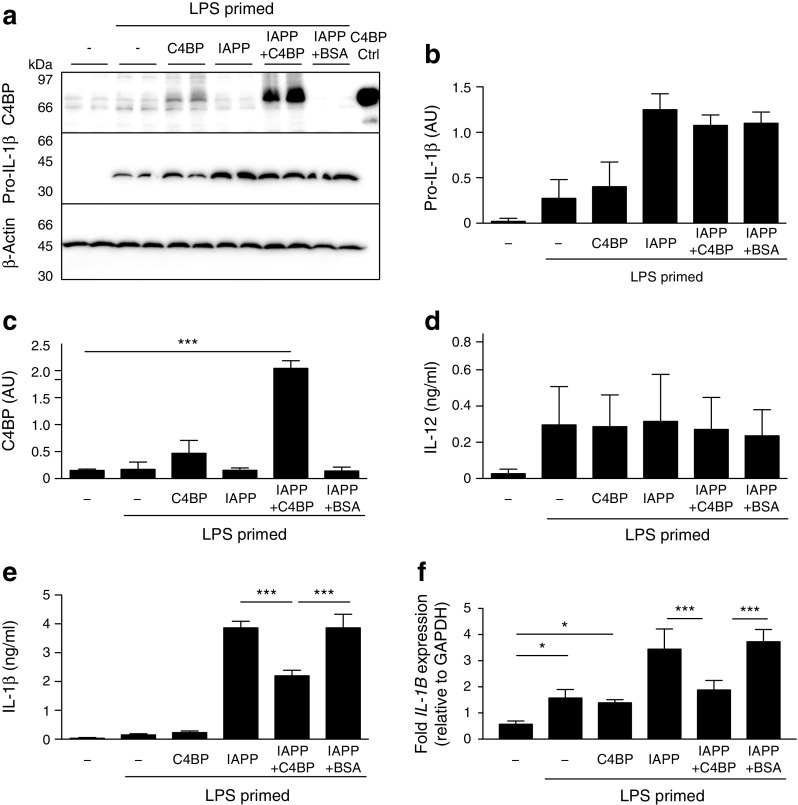
C4BP does not affect inflammasome priming but is internalised by macrophages in the presence of IAPP. (**a**) Western blot for C4BP uptake and pro-IL-1β expression in THP1 cell lysates. (**b**) Densitometry results for lysate pro-IL-1β. (**c**) Densitometry results for lysate C4BP. (**d**) ELISA measurement of IL-12 in supernatant fraction. (**e**) IL-1β secretion from the same cells as measured by ELISA. (**f**) Quantitative PCR analysis of *IL-1Β* expression in treated THP1 cells. AU, arbitrary units. *p < 0.05, ***p < 0.001 as indicated. Ctrl, control

To determine whether inhibition of IL-1β secretion was caused by decreased expression of pro-IL-1β, lysates and supernatant fractions of THP1 cells incubated overnight with or without IAPP and/or C4BP were analysed. LPS-primed cells upregulated pro-IL-1β, which was further upregulated by IAPP addition but was unaffected by C4BP (Fig. 3a, b). C4BP was only found within cell lysates when added in the presence of IAPP (Fig. 3a, c), indicating cellular binding or uptake facilitated by IAPP. Addition of C4BP did not affect LPS-induced secretion of IL-12 (Fig. 3d) or IL-6 (not shown), but still inhibited IAPP-induced IL-1β secretion from the same cells (Fig. 3e). C4BP did not affect *IL1B* mRNA levels within LPS-primed cells when added alone, but did inhibit the further increase in pro-IL-1β expression mediated by IAPP (Fig. 3b, f).

### C4BP and IAPP co-localise and are taken up as a complex

**Fig. 4:**
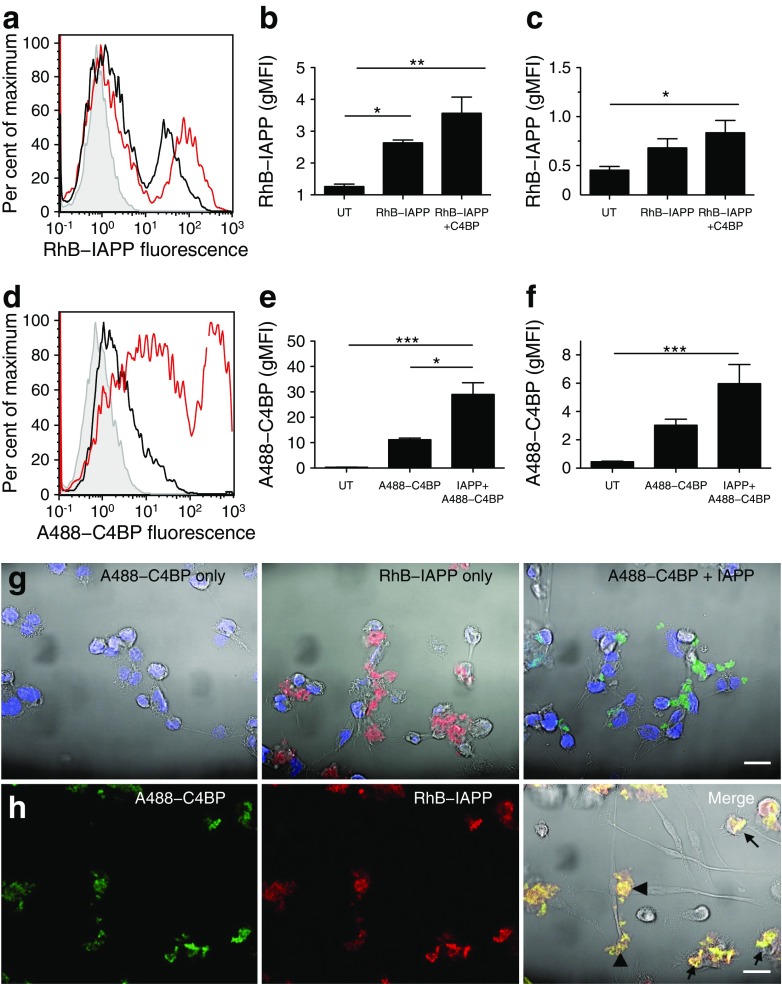
C4BP is internalised together with IAPP. (**a**) RhB–IAPP (25 μmol/l) was incubated with phorbol 12-myristate 13-acetate-differentiated THP1 cells overnight, and cellular uptake was assessed by flow cytometry. Grey line, untreated cells; black line, cells+RhB–IAPP; red line, cells+RhB–IAPP+C4BP. (**b**) Quantification of THP1 IAPP uptake. (**c**) Quantification of MDM IAPP uptake. (**d**) Flow cytometric analysis of A488–C4BP uptake, in the presence or absence of IAPP. Grey line, untreated cells; black line, cells+A488–C4BP; red, cells+A488–C4BP+IAPP. (**e**) Quantification of THP1 C4BP uptake. (**f**) Quantification of MDM C4BP uptake. UT, untreated. (**g**) Confocal analysis of THP1 cells with A488–C4BP (green) or RhB–IAPP (red). DAPI, blue. Aggregates were always found in association with cells. (**h**) Confocal images showing A488–C4BP (green) and RhB–IAPP (red), which strongly co-localise and form complexes at MDM cell surfaces (arrowheads) and intracellularly (arrows). Yellow, co-localisation. Scale bars, 20 μm. *p < 0.05, **p < 0.01 and ***p < 0.001 as indicated. gMFI, geometric mean fluorescence intensity

To further investigate C4BP–IAPP interactions, we fluorescently labelled C4BP (A488–C4BP) and IAPP (RhB–IAPP). Uptake of RhB–IAPP was enhanced by the presence of C4BP in THP1 cells (Fig. 4a, b) and MDMs (Fig. 4c). A488–C4BP was only significantly taken up in the co-presence of IAPP (Fig. 4d, e). Similar results were found for THP1 cells and MDMs (Fig. 4f). These results were confirmed by confocal microscopy: added alone, A488–C4BP did not associate with THP1 cells (Fig. 4g), while RhB–IAPP formed fibrillar amyloid aggregates. When added together, the two proteins strongly co-localised in insoluble aggregates (Fig. 4g). Similar results were found with MDMs, where C4BP and IAPP added together formed complexes both at the cell surface and intracellularly (Fig. 4h).

### C4BP–IAPP complexes localise to phagolysosomal compartments

**Fig. 5:**
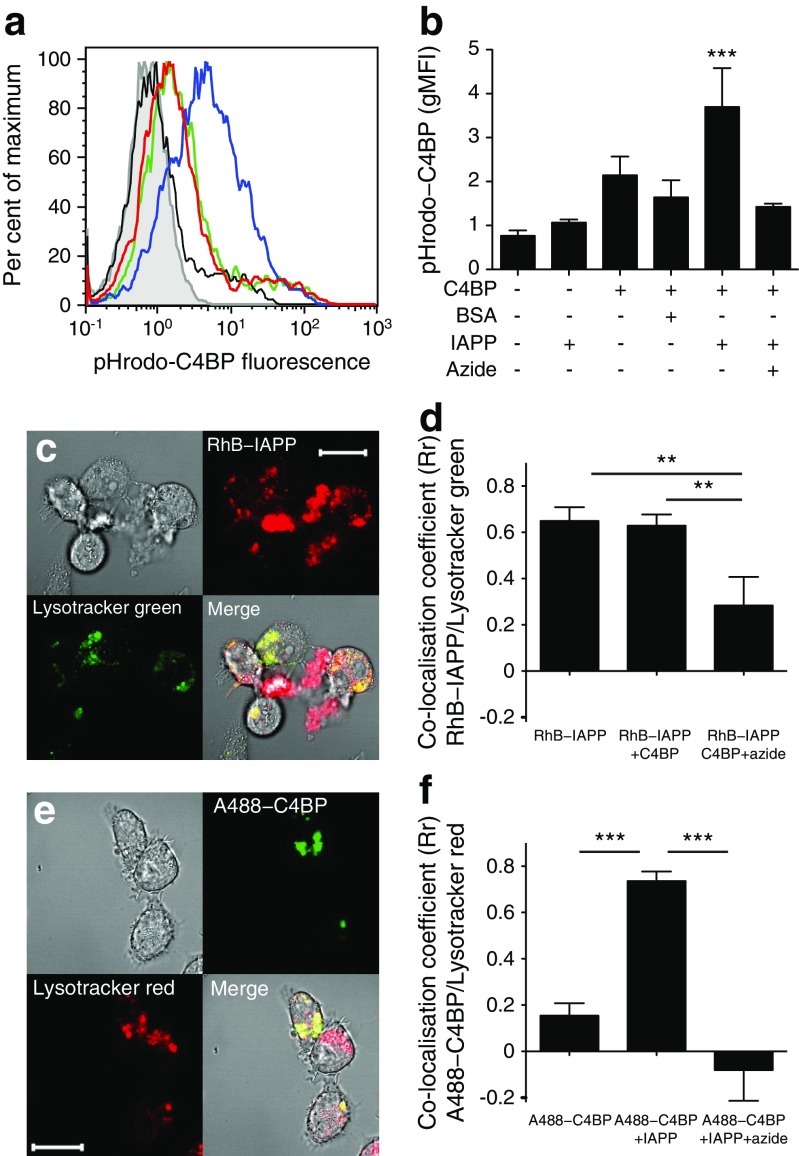
IAPP–C4BP complexes are internalised to lysosomal compartments. (**a**) Flow cytometric assessment of pHrodo-C4BP uptake with or without IAPP. Grey line, untreated cells; black line, +IAPP; green line, +pHrodo–C4BP; red line, +pHrodo–C4BP+BSA; blue line, +pHrodo–C4BP+IAPP. (**b**) Quantification of the pHrodo–C4BP uptake by THP1 cells shown in (**a**). (**c**) Confocal microscopy assessment of RhB–IAPP co-localisation with Lysotracker green within THP1 cells. (**d**) Quantification of co-localisation shown in (**c**) with or without C4BP. (**e**) A488–C4BP co-localisation with Lysotracker red, in the presence of unlabelled IAPP. (**f**) Quantification of the co-localisation shown in (**e**) with or without unlabelled IAPP. Similar results were seen in MDMs (not shown). Scale bars, 20 μm. **p < 0.01 and ***p < 0.001 as indicated; or compared with untreated cells in (**b**)

To further investigate internalisation, C4BP was labelled with pHrodo, which fluoresces in acidic compartments. Addition of pHrodo–C4BP to THP1 cells resulted in increased fluorescence only in the presence of IAPP (Fig. 5a, b). Similar results were found with MDMs (not shown). Internalised IAPP co-localised strongly with Lysotracker-stained lysosomal compartments (*R* ^2^ = 0.649) (Fig. 5c). This was unaffected by C4BP (Fig. 5d), although uptake was inhibited by azide. In contrast, C4BP only localised to lysosomal compartments when IAPP was present (Fig. 5e, f). Similar results were found using MDMs (not shown).

### C4BP preserves phagolysosomal integrity after IAPP uptake

**Fig. 6:**
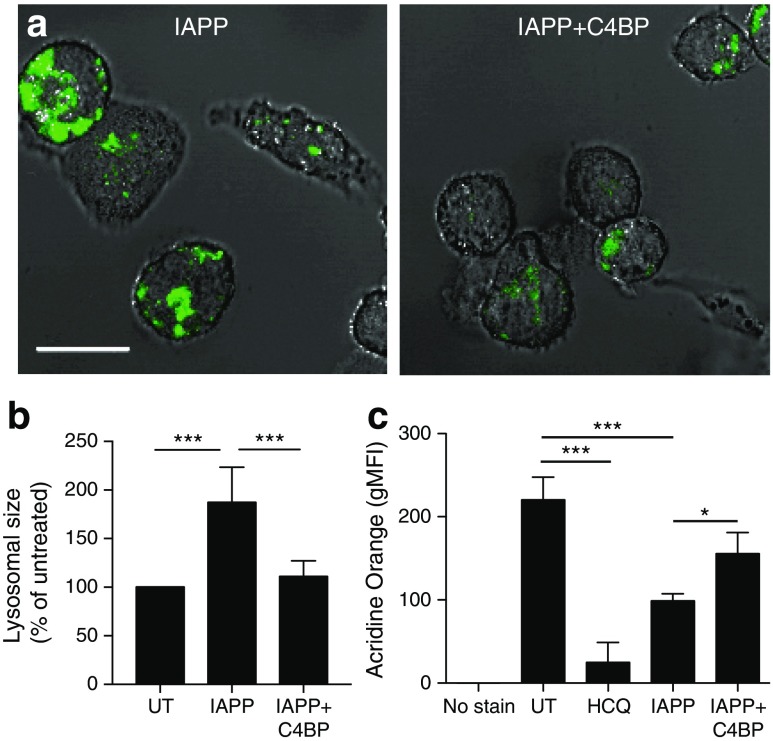
C4BP prevents IAPP-mediated loss of lysosomal integrity. (**a**) THP1 cells incubated overnight with 10 μmol/l IAPP with or without 0.5 μmol/l C4BP were stained with Lysotracker dye and analysed by confocal microscopy. Images are representative of five repeats. Scale bar, 20 μm. (**b**) Quantification of data from (**a**) showing average lysosomal size per cell. UT, untreated. (**c**) Intact lysosomes in MDMs incubated for 8 h with hydroxychloroquine (HCQ) control, 20 μmol/l IAPP, or 20 μmol/l IAPP + 0.6 μmol/l C4BP were stained using Acridine Orange and analysed by flow cytometry. *p < 0.05, ***p < 0.001 as indicated

Although C4BP does not inhibit IAPP uptake, it may inhibit inflammasome activation by preventing IAPP-mediated destabilisation of lysosomal membranes. To address this, we analysed images of Lysotracker-stained THP1 cells incubated with IAPP with or without C4BP. Cells incubated with IAPP alone had significantly larger swollen lysosomes, typical of particulate-induced instability leading to NLRP3 inflammasome activation [19]; this was reversed by C4BP (Fig. 6a, b). Cells were also stained with Acridine Orange, a lysosomotropic pH-sensitive metachromatic dye used to study lysosomal membrane integrity. As control, de-acidification of lysosomes with hydroxychloroquine led to a significant loss of fluorescence (Fig. 6c). Cells incubated with IAPP displayed a similar decrease, which was significantly inhibited by C4BP (Fig. 6c). Together, these results show that C4BP prevents IAPP-mediated destabilisation of the phagolysosomal membrane.

### C4BP-mediated IL-1β inhibition preserves beta cell function

**Fig. 7:**
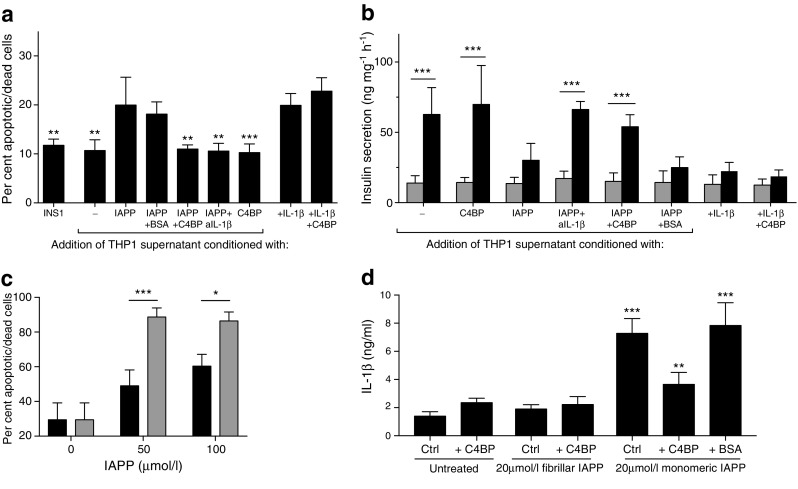
C4BP inhibition of IAPP-mediated inflammasome activation preserves beta cell viability and function. (**a**) INS-1 viability after addition of THP1 supernatant fraction, as tested by flow cytometry after Annexin V staining. (**b**) Insulin secretion from INS-1 cells exposed to THP1 supernatant fractions, as measured by ELISA. Grey bars, low glucose; black bars, high glucose. (**c**) Viability of INS-1 cells incubated overnight with freshly prepared monomeric IAPP (grey bars) or with pre-formed IAPP fibrils (black bars). (**d**) IL-1β secretion from LPS-primed THP1 cells stimulated with IAPP monomers or pre-formed IAPP fibrils. aIL-1β, anti-IL-1β antibody. *p < 0.05, **p < 0.01 and ***p < 0.001 as indicated; or compared with IAPP-conditioned supernatants in (**a**), or compared with untreated control cells (Ctrl) in (**d**)

To validate the protective effect of C4BP, supernatant fractions from THP1 cells were incubated with INS-1 clonal beta cells overnight. Supernatant fractions from IAPP-incubated THP1 cells induced a moderate but significant increase in apoptosis, which was reversed by co-incubation with C4BP (Fig. 7a). Apoptosis was blocked by neutralising anti-IL-1β antibody and recapitulated by addition of recombinant IL-1β, demonstrating IL-1β dependence. Although apoptosis induction was moderate, glucose-stimulated insulin secretion (GSIS) was drastically inhibited by IAPP (Fig. 7b). This process was also shown to be IL-1β dependent, as it was reversed by IL-1β-neutralising antibody. Again, the presence of C4BP during incubation of THP1 cells with IAPP prevented IL-1β-dependent GSIS loss. Addition of C4BP to recombinant IL-1β did not inhibit apoptosis or GSIS loss, confirming that C4BP does not inhibit IL-1β directly. Supernatant fractions incubated overnight with IAPP contain mature amyloid fibrils. To ensure that these did not induce cell death, pre-incubated fibrils or fresh IAPP monomers were incubated with INS-1 cells overnight and cell death was measured (Fig. 7c). Mature fibrils caused significantly less apoptosis compared with monomers. Consistent with their inability to permeabilise membranes, pre-formed amyloid fibrils were also unable to induce inflammasome activation (Fig. 7d). These results indicate that IL-1β is the sole component of IAPP-induced THP1 supernatant fraction responsible for inhibiting beta cell survival and function and that C4BP acts at the level of inflammasome activation to protect beta cell function.

### Human pancreatic islet cells express and secrete C4BP

**Fig. 8:**
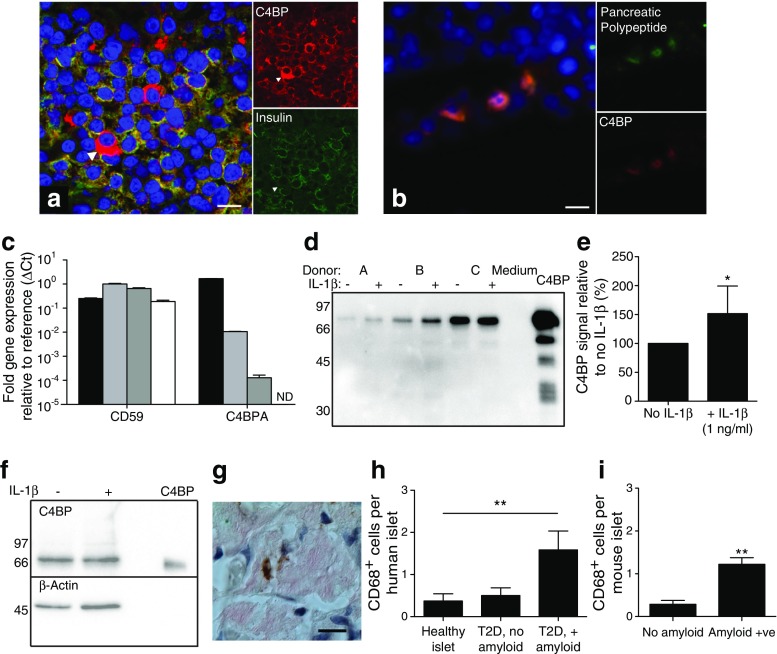
C4BP is expressed in human pancreatic islets. (**a**) Human pancreatic islets stained for C4BP (red) and insulin (green). Arrowhead shows a cell strongly positive for C4BP but negative for insulin. Scale bars 10 μm. (**b**) Co-localisation of C4BP with pancreatic polypeptide. Scale bar, 10 μm. (**c**) Quantitative PCR detection of *C4BPA* expression in RNA from purified human pancreatic islets (light grey), liver (black), HepG2 cells (dark grey) and MDMs (white). Expression of *CD59* mRNA was control. ND, not detected. (**d**) Overnight supernatant fractions from isolated human pancreatic islets were blotted for C4BP α-chain. Blot is representative of two experiments, using a total of five donors. (**e**) Densitometry quantification results showing an IL-1β-induced increase in C4BP secretion as detected by western blot, from a total of five donors. (**f**) Western blot for C4BP in human pancreatic islet lysates, representative of three repeats. (**g**) Human pancreas sections from individuals with type 2 diabetes or from healthy control individuals were stained for amyloid deposits (Congo Red) and macrophage marker CD68 (brown). Scale bar, 20 μm. (**h**, **i**) Results of CD68 and Congo Red staining in human and hIAPP transgenic mouse islets, respectively. T2D, type 2 diabetes. Statistics in (**e**) and (**i**), *t* test. Statistics in (**h**), comparing amyloid vs no amyloid. *p < 0.05, **p < 0.01 as indicated

As it has been suggested that extrahepatic local complement protein production is important in inflammation [20], we investigated islet C4BP production. Human islet staining primarily showed C4BP in insulin-positive beta cells (Fig. 8a). A minority population of strongly expressing cells was identified. These cells were negative for glucagon, insulin, somatostatin and CD68 (not shown) but were positive for pancreatic polypeptide (Fig. 8b). Quantitative PCR revealed that human liver contained the highest levels of C4BP transcript, although pancreatic islet expression was still 100-fold higher than in HepG2 hepatocarcinoma cells (Fig. 8c). C4BP secretion from isolated human islets was confirmed by western blot (Fig. 8d) and its secretion increased upon IL-1β addition (Fig. 8d, e), showing that human islets respond to inflammasome-derived inflammation by increasing C4BP release. The presence of C4BP in islet lysates was also confirmed (Fig. 8f). In pancreatic sections from individuals with type 2 diabetes, islets with amyloid deposits contained significantly more macrophages than those without (Fig. 8g, h), reflecting the association between IAPP, macrophages and inflammation. No islets from healthy donors contained amyloid deposits. In contrast to humans, rodent IAPP does not form amyloid [21]. However, human IAPP (hIAPP) transgenic rodents develop amyloid deposits and type 2 diabetes-like disease [22]. Consistent with human data, amyloid-positive hIAPP mouse islets had increased CD68^+^ infiltrating cells (Fig. 8i).

## Discussion

We have found that human C4BP is secreted from isolated human pancreatic islets and inhibits IAPP-mediated inflammasome activation and secretion of the diabetogenic cytokine, IL-1β. C4BP is a 500 kDa multimer, with seven identical α-chains and one β-chain, forming an ‘octopus’-like structure. C4BP is unusual among complement proteins in that there is no reported human deficiency [23], suggesting an important conserved role outside traditional complement regulation. By binding to apoptotic cells and regulating complement activation, C4BP is involved in the non-inflammatory clearance of cellular debris [24]. Here, we describe a novel mechanism whereby C4BP also maintains ‘silent’ clearance of endogenous material. Previously, we identified IAPP binding sites on C4BP α-chain domains 2 and 8 [11]. C4BP allowed increased IAPP uptake by macrophages but did not alter localisation of IAPP to phagolysosomes. However, C4BP did prevent phagolysosomal membrane destabilisation, a recognised mechanism of particulate-mediated inflammasome activation [10, 19]. Current models indicate that active nucleation of amyloid is required for IAPP-mediated membrane disruption, while mature fibrils do not cause loss of barrier function [8, 9, 25], consistent with our findings that mature fibrils caused limited toxicity and no inflammasome activation. This explains how the observed C4BP-mediated inhibition of fibrillation limits the proinflammatory potential of IAPP. However, we cannot rule out that C4BP also ‘coats’ IAPP aggregates and limits their membrane interactions, as C4BP also limited inflammasome activation by MSU and SiO_2_ crystals. Using IAPP from a different supplier under differing buffer conditions, we previously described that C4BP was able to enhance fibril formation, but we now show convincing evidence that C4BP inhibits IAPP fibrillation when tested in cell culture conditions. Preliminary results using recombinantly expressed IAPP were consistent with this action of C4BP. Further studies are required to elucidate in detail the effects of C4BP on the kinetics of IAPP fibril formation under various experimental conditions.

The role of IL-1β in type 2 diabetes was demonstrated by clinical trials using the recombinant IL-1 receptor antagonist anakinra: it improved glycaemic control in individuals with type 2 diabetes [4] and improved inflammation and beta cell function for months after therapy cessation [3]. In animal models, targeting IL-1β decreased islet inflammation and improved glycaemic control [2, 26]. Islet macrophage infiltration and expression of inflammasome components and pro-IL-1β are increased in type 2 diabetes [1]. Beta cells express extremely high levels of the IL-1 receptor [27] and we and others show that IL-1β leads to beta cell dysfunction [28–32]. The expression of C4BP within the human islet, the site of IAPP and IL-1β production, is therefore of interest as a local mechanism of inflammasome regulation. C4BP α-chain expression is highest in liver, the main source of circulating serum C4BP, but was also high in isolated islets. In comparison, CD59, a ubiquitously expressed cell surface complement inhibitor, was comparably expressed in all tested tissues/cells, although highest in pancreatic islets, where it is required for insulin secretion [33]. IL-1β increases C4BP secretion from isolated islets, and serum C4BP levels are also increased in diabetic individuals [34, 35]. Locally produced C4BP could therefore act in islet homeostasis, protecting beta cells against the deleterious effects of IAPP. Accordingly, C4BP co-localises with IAPP amyloid in vivo in pancreatic islets from humans with type 2 diabetes [11]. Further work with *C4bp*-knockout mice will be needed to fully determine how C4BP affects IAPP-mediated inflammation and amyloid deposition in pancreatic islets in situ.

C4BP also interacts with other amyloidogenic proteins such as Aβ peptide [36] and neocortical plaques in individuals with Alzheimer’s disease [37]. We have also described binding of C4BP to the truncated form of thioredoxin, Trx80 [38], which forms aggregate deposits in human brains [39] and have shown that C4BP binds to Aβ peptide [40], prion β-oligomers and fibrils [41]. In Alzheimer’s disease, C4BP binds to amyloid deposits and local C4BP expression is also upregulated [40]. The implications for protective effects of C4BP in amyloid-mediated diseases are clear, especially as inflammasome activation is proposed as a common disease mechanism in many amyloid- or protein aggregate-induced diseases [12, 13]. We propose that C4BP could function as an extracellular chaperone protein for neutralisation of amyloidogenic proteins in human diseases.

## Electronic supplementary material

ESM Methods (PDF 333 kb)

## Abbreviations

A488: Alexa-Fluor 488
AIM2: Absent in melanoma 2
C4BP: C4b binding protein
GSIS: Glucose-stimulated insulin secretion
IAPP: Islet amyloid polypeptide
hIAPP: Human IAPP
LPS: Lipopolysaccharide
mC4BP: Monomeric C4BP
MDM: Monocyte-derived macrophage
MSU: Monosodium urate
NLRP3: NOD-like receptor pyrin domain containing-3
RhB: Rhodamine B
ThT: Thioflavin T
