# Supplementary material for: The human serum protein C4b-binding protein inhibits pancreatic IAPP-induced inflammasome activation
Source: Diabetologia. 2017 May 12;60(8):1522–33. doi: 10.1007/s00125-017-4286-3 (PMC5491568; doi:10.1007/s00125-017-4286-3)
Supplement: Supplementary file 1 — (PDF 333 kb) [file 125_2017_4286_MOESM1_ESM.pdf]

Electronic Supplementary Methods

Cells: THP1 cells (ATCC) were maintained in RPMI-1640 (Gibco) containing 10% (vol/vol) FCS, penicillin, and streptomycin. INS-1 832/13 cells were grown in the same medium additionally supplemented with 10 mmol/l HEPES (Sigma), 2 mmol/l L-glutamine, 1 mmol/l sodium pyruvate (Gibco) and 50 μmol/l -2 mercaptoethanol (Sigma). Human monocyte-derived macrophages (MDMs) were produced by incubating freshly purified human PBMCs in tissue culture flasks in macrophage serum free medium (M-SFM) (Gibco) for 1 hr, followed by washing and differentiation for 1 week in M-SFM with 50 ng/ml GM-CSF (Immunotools). Differentiation was determined by CD11b expression using a Cyflow flow cytometer (Partec). Human PBMC were obtained from consenting informed healthy donors under permit 2013/846 approved by the Regional Ethics Committee in Lund. All cells were tested monthly for mycoplasma contamination and were found to be negative.

Inflammasome activation assay: THP1 cells were plated with 250 nmol/l phorbol 12-myristate 13-acetate (PMA) (Sigma) for 2 hrs, then medium exchanged and cells differentiated overnight. Cells were primed with 500 ng/ml lipopolysaccharide (LPS) (Sigma) for 3 hrs, washed three times in PBS, and 150 μl Opti-MEM added containing 200 μg/ml monosodium urate crystals (MSU) (Invivogen), or 25 μmol/l IAPP, in presence or absence of up to 0.6 μmol/l (300 g/ml) C4BP, or control proteins (300 g/ml). Unless otherwise indicated, MSU was incubated for 1 hr, and IAPP overnight. Other inflammasome activators included 5 mmol/l ATP (Sigma), 20 mmol/l H_2O_2, 7.5 μg/ml Imiquimod (Invivogen), 10 μg/ml silica nanoparticles (SiO_2) (Invivogen) or transfection

with 5 μg/ml poly(deoxyadenylic-deoxythymidylic) acid (poly(dA:dT)) (Invivogen) and Lipofectamine 2000 (Invitrogen). Antibody specificity was verified using non-immune serum.

Cytokine detection: Secreted cytokines were detected by ELISA (Mabtech, Peprotech), or using IL-1β-specific HEK-Blue reporter cells (Invivogen), according to supplier's instructions. Briefly, THP1 supernatant was added to HEK-Blue cells and incubated overnight, and alkaline phosphatase secreted in response to IL-1β was detected using QUANTI-Blue reagent (Invivogen) and reading the developed colour at 620 nm. IL-1β specificity was determined by neutralising mouse IgA anti-IL-1β antibody (Invivogen). Caspase-1 was detected using the Caspase-Glo 1 assay (Promega), and specificity assessed using the caspase-1 specific inhibitor Ac-YVAD-CHO, according to instructions.

IAPP/C4BP uptake assays: MDMs or PMA-differentiated THP-1 cells were plated for 24 hrs, before replacing medium with M-SFM or optiMEM respectively with or without 25 μmol/l IAPP and 0.5 μmol/l C4BP. After overnight incubation, cells were PBS washed, then harvested with trypsin. Uptake of A488- or pHrodo-labelled C4BP, or Rhodamine-labelled IAPP (RhB-IAPP), was assessed by flow cytometry. For confocal microscopy, cells were plated in 6-lane tissue culture microscope slides (Ibidi). After incubation, cells were washed twice with PBS, fixed with 3.7% PFA for 10 min, permeabilized with PBS+0.1% Triton X-100 for 5 min, stained with 300 nmol/l DAPI (Invitrogen) for 5 min before mounting with Fluorescent Mounting Medium (DAKO). Alternatively, cells were incubated 4 hrs, PBS washed twice, then stained with 75 nmol/l lysotracker reagents

(Molecular Probes). Cells were imaged using a Zeiss LSM 510 Meta Confocal microscope with 63x oil lens. Images were processed using Zen software. Colocalization was calculated using Colocalizer Pro software, analysing multiple cells from at least 5 images from each of 3 individual experiments. Average cellular lysosomal size was analysed using ImageJ, analysing at least 50 cells from each of 5 individual experiments.

Viability and insulin secretion: INS-1 cells were plated overnight then growth medium replaced with 66 μl fresh medium plus 33 μl supernatant from THP-1 cells activated as described above. Recombinant human IL-1β (Mabtech) was used at final concentration of 1.5 ng/ml, C4BP at 0.6 μmol/l, and IL-1β neutralizing IgA antibody (InvivoGen) at 2 μg/ml. After 24 hrs, cell death was measured by flow cytometry using Annexin V- APC (ImmunoTools) and 7-AAD Viaprobe (BD Biosciences). For insulin secretion, INS-1 cells were pre-incubated in HBSS with 2.8 mmol/l glucose for 2 hrs before incubation for 1 hr in HBSS containing 2.8 or 16.7 mmol/l glucose. Insulin was measured by ELISA (Mercodia) and normalized to total lysate protein content as measured by BCA assay (Thermo Scientific). Secretion is expressed as ng insulin per mg total protein per hour (ng mg^{-1} hr^{-1}).

Islet staining: Formalin-fixed paraffin-embedded pancreas sections from five T2D and five non-diabetic patients were deparaffinized and heat-treated in 10 mmol/l sodium citrate for 20 minutes, then immunolabelled with mouse monoclonal anti-CD68/SR-D1 (Novus Biological), diluted 1:10, overnight at RT. Frozen pancreas sections from six hIAPP transgenic male mice (12-16 months old) were fixed in 10% wt/vol neutral buffered formalin for 10 minutes then incubated in CD68/SR-D1, diluted 1:10, overnight at RT. In connection with the assessment of CD68 reactivity, sections were stained for

amyloid with Congo red. For C4BP staining, frozen human pancreas samples were cut in 8 μm slices, incubated in 2% wt/vol paraformaldehyde for 10 minutes then 100% ethanol for 5 minutes, washed in TBS and then stained overnight at 4°C with 1:250 guinea pig anti-insulin (DAKO), 1:50 mouse monoclonal anti-C4BP (in–house), or 1:250 sheep anti-pancreatic polypeptide (SeroTec), followed by 2 hr incubation with highly absorbed A488-labelled goat anti-rabbit or goat anti-guinea pig, or donkey anti-sheep (all 1:1000, Invitrogen), or 1:1000 Alexa546-labelled goat anti- mouse (Invitrogen). Nuclei were identified using DAPI, and the results were studied in a confocal microscope (Zeiss LSM 780). Isotype controls and exclusion of primary antibodies resulted in complete removal of staining.

Data inclusion and blinding: All data was included in the study, no values were excluded. Values were not randomized; experimenters were not blinded.
